# Predictive Value of Clinical Judgment of Tumour Progression in Phase II Trials

**DOI:** 10.1371/journal.pone.0052638

**Published:** 2012-12-26

**Authors:** Nuria Kotecki, Nicolas Penel, Antoine Adenis, Charles Ferte, Stéphanie Clisant

**Affiliations:** 1 Centre Oscar Lambret, Lille, France; 2 Lille-Nord-de-France University (EA 2694), Lille, France; Robert Wood Johnson Medical School, United States of America

## Abstract

**Background:**

The diagnosis of tumour progression or progressive disease (PD) is a key element for designing and interpreting contemporary phase II trials. In some cases, PD is stated by the physician and is not formally confirmed by imaging.

**Purpose:**

In this study, we intend to analyze the value of the PD based on clinical judgment and the risk of overestimating the occurrence of PD by clinical judgment.

**Methods:**

We have conducted a single-centre retrospective study to analyse the diagnostic accuracy of this clinical judgment compared to planned imaging including all patients enrolled in our institution in phase II trials investigating systemic treatments for advanced solid tumours between January 2008 and November 2010.

**Results:**

The positive predictive value (PPV) and the specificity of clinical judgment of PD was very high (>90%).

**Conclusions:**

According to this study, the clinical judgment of PD is highly predictive of radiological PD as assessed, for example, by RECIST. Physicians do not overestimate PD occurence. Clinical judgment of PD could be taken into account in the definition of PD.

## Introduction

Phase II trials are designed to sort out drugs with disappointing level of activity. The decision rules and sample size calculation of phase II trials are basically based on the following parameters: P0 (an inacceptable level of activity, “failure rate”), P1 (a desirable level of activity, “success rate”) and the couple α/β [1]. At the end, the primary endpoint is used as a binary parameter that partitions patients into two categories: responders (success) and non-responders (failure).

Regardless of the method used for assessing the activity of new drugs or new regimens in phase II trials (objective response rates [2], [3], non-progression rate at fixed time points [4], growth modulation index [5], etc.) tumour progression (or progressive disease, PD) is a key element for defining success or failure. As per protocol and to the extent possible, investigators should document tumour progression by imaging. This assessment, however, is not possible in some circumstances: rapid deterioration of the patient's general condition, contraindication of imaging, refusal of a new examination, patient withdrawal of informed consent … These circumstances are taken into account in guidelines defining PD in clinical trial, such as RECIST [2]. RECIST integrates clinical judgement of PD, when imaging is not feasible or possible [2]. Nevertheless, in case of clinical judgment of PD without confirmation by imaging, there are doubts concerning the robustness and reliability of this information. Is the clinical judgment of PD really predictive of progression subsequently proven by imaging? Furthermore, is there an overestimation of PD occurrence by physicians? Finally, do severe toxicities altering the patient general conditions mimic PD? We have reviewed all consecutive medical records of patients treated in phase II trials in our institution in order to explore the predictive value of clinical judgment of PD.

## Patients and Methods

### General methodology

An independent investigator (NK) reviewed the medical records of all consecutive patients enrolled in phase II trials investigating systemic treatment for advanced solid tumours between January 2008 and November 2010. The data collected were (i) tumour and patient characteristics at baseline, (ii) the nature of investigational agents or combinations, (iii) tumour status at the last assessment, (iv) clinical judgment of progression and (v) results of planned tumour imaging.

Diagnostic accuracy of the clinical judgment of PD was examined as a diagnostic test in comparison to PD diagnosed by imaging according to RECIST [2]. We constructed a classical 2×2 table in order to calculate sensitivity (Se), specificity (Sp), positive predictive value (PPV), negative predictive value (NPV) and accuracy (the rate of well-classified patients). Comparisons between the different categories of patients were conducted using the Fischer exact test and the Mann-Whitney test. Survival curves were constructed using the Kaplan-Meier method and comparisons were carried out with the log rank test.

### Ethics

The internal ethic board of our institution (Clinical Trial Commission; “Commission interne des études cliniques”) had approved this study. According to the French laws (law of the 06th January 1978 about data, data-collection and freedom, in case of single-centre, retrospective study based on already recorded and stored data, there is no need of specific written informed consent; but all patients have been orally informed about the potential use of their collected data for future research. We have obtained the agreement N° 1034071 from the “National Commission about Data-collection and Freedom” (“Commission Nationale Informatique et Liberté”).

## Results

### General

129 patients were included in 32 different phase II trials between January 2008 and November 2010. Until now, 84 (65%) patients discontinued the investigational treatment for PD; 27 discontinued the trial treatment for reasons other than PD: investigator's decision (2 cases), severe toxicities (13 cases), consent withdrawal (3 cases), death from other cause (2 cases) and study termination (7 cases).18 patients are still currently treated in these phase II trials. Patient characteristics are listed in Table 1.

**Table 1:** Baseline characteristics.

Parameters	No. (%)
Primaries	
Breast cancer	14 (16.0)
Non small cell lung cancer	13 (15.5)
Sarcoma	13 (15.5)
Colorectal cancer	11 (13.0)
Prostate cancer	8 (9.5)
Head and neck cancer	5 (6.0)
Pancreas cancer	5 (6.0)
Gastro-intestinal stromal tumour	4 (4.5)
Mesothelioma	4 (4.5)
Ovary cancer	3 (3.5)
Renal cell cancer	3 (3.5)
Carcinoma of unknown primary	2 (2.0)
WHO-Performance status	
1	36 (43.0)
2	46 (54.5)
3	2 (2.5)
Treatment under investigation	
Chemotherapy	17 (20.0)
Molecular targeted therapy	39 (46.5)
Chemotherapy+Molecular targeted therapy	28 (33.5)

### Description of tumour progressions

**Figure 1:**
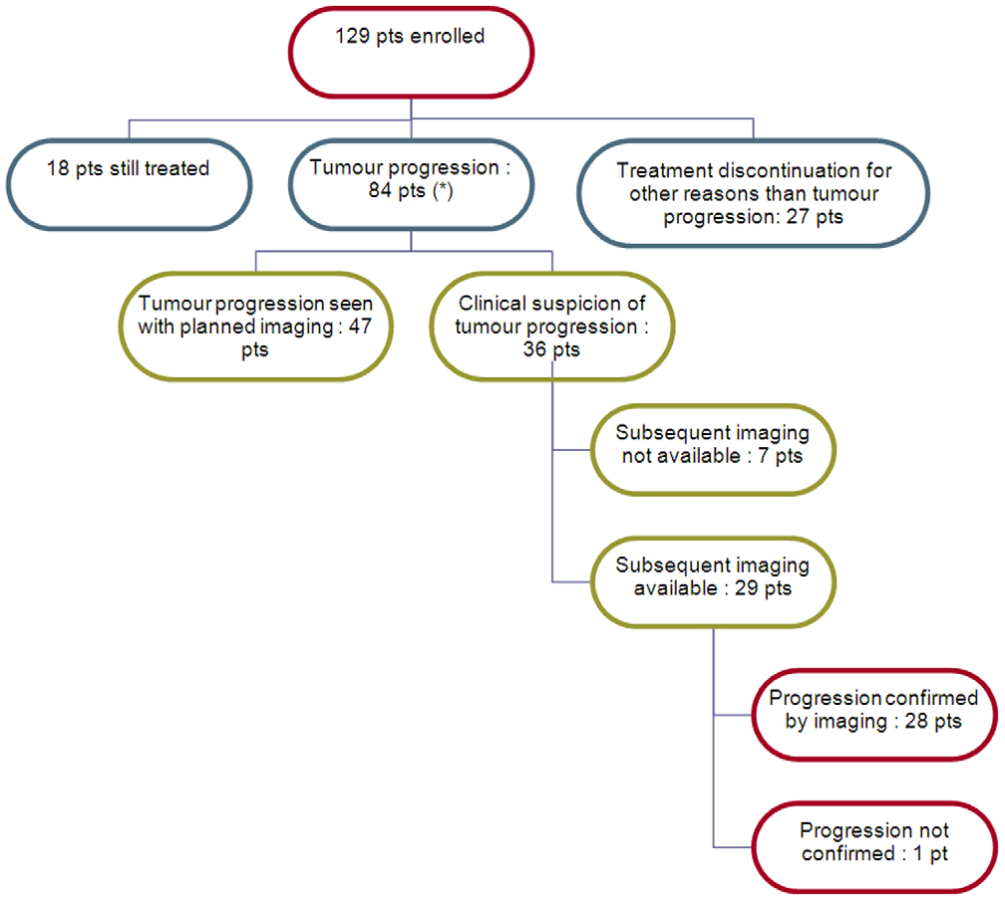
Partition of patients. (*) one patient excluded because progression was defined by increased in tumor marker. Pt: patient.

Eighty-four patients experienced PD with the trial treatment, among whom 47 PD were documented by planned imaging without clinical signs suggestive of tumour progression (**Figure 1**). One patient experienced “biological progression” without radiological confirmation (ovarian cancer patient with increasing CA125; this patient was excluded from subsequent analysis). “Clinical judgment of PD” was pronounced for 36 patients, among whom imaging was not available in 7 cases (withdrawal of informed consent (3 cases), death in the very next days (3 cases) or severe deterioration of general condition (1 case). Imaging was available in the other 29 cases and PD was confirmed in 28 of them (Table 2).

**Table 2:** Performance of clinical judgment of tumour progression.

	Progressive disease according to RECIST	Absence of progression according to RECIST
Clinical judgment of tumour progression	28	1
Absence of clinical sign of progression	47	18
Parameter	%	95%-Confidence intervals
Sensitivity	37	26–48
Specificity	94	84–100
Positive predictive value	96	89–100
Negative predictive value	27	16–38
Accuracy	49	38–59

### Diagnostic accuracy of clinical judgment of progression

The PPV of clinical judgment of PD was very high (96%). In other words, when physicians suspected tumour progression, it was confirmed by subsequent imaging in the vast majority of cases. The specificity of clinical judgment was also high (94%), meaning that radiological examination rejected only very few clinical judgments of PD. On the contrary and as expected, because most of PD are asymptomatic, the Se and NPV were low, at 37% and 27% (Table 2).

### Factors associated with clinical judgment of tumour progression

**Figure 2:**
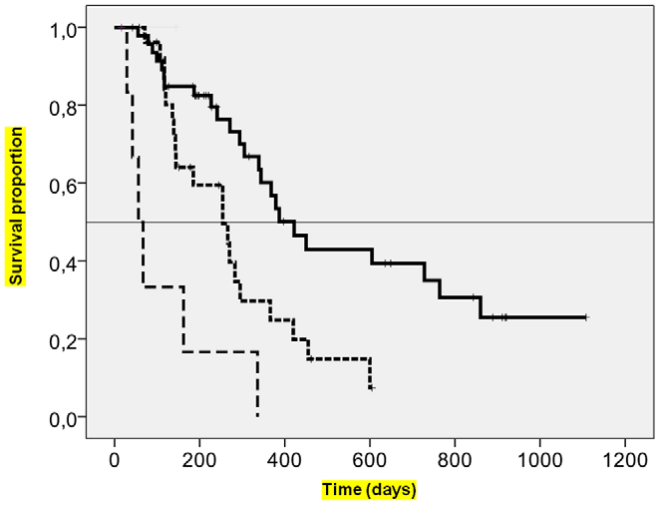
Overall survival from the date of progression. Plain line: overall survival of patients with tumour progression diagnosed by planned imaging (n = 47; median overall survival: 441 days). Dotted-line: overall survival of patients with clinical judgment of tumour progression confirmed by subsequent imaging (n = 28; median overall survival: 285 days). Broken-line: overall survival of patients with clinical judgment of progression without available imaging: (n = 7; median overall survival: 56 days). p<0.0001.

The sole clinical factor different among patients with clinical judgment of PD and those with PD diagnosed by planned tumour assessment was the deterioration of general patient status (Table 3). The median time to PD was 118 days for patients with progression diagnosed by planned imaging, 102 days for patients with clinical judgment of PD confirmed by subsequent imaging, and 28 days for patients with clinical judgment of PD without imaging confirmation (p<0.0001). This suggests that physicians reach an early diagnosis of symptomatic forms of PD and then anticipate tumour assessment. The overall survival of patients with clinical judgment of PD without radiological documentation was worse than that of patients with radiological documentation of PD (p<0.05) (**Figure 2**). Moreover, at the time of PD, maximal haematological and non-haematological toxicities were similar in patients with clinical judgment of PD, whether or not radiological documentation was available (Table 3). This suggests that treatment-related toxicities did not mimic PD.

**Table 3:** Patient characteristics at progression.

Parameter		Clinical judgment of tumour progression with radiological confirmation (*n = 28*)	Clinical judgment of tumour progression without radiological confirmation (*n = 7*)	*p*
WHO-performance status at progression	Median	1	3	
	Extreme values	0–4	1–4	0.030
Maximal grade of hematological toxicity	Median	2	2	
	Extreme values	0–2	0–2	0.510
Maximal grade of non-hematological toxicity	Median	3	3	
	Extreme values	2–4	2–4	0.560

### Limitations

There are several limitations related to the retrospective and single-centre nature of this study. For example, in seven cases of patients with clinical judgment of PD, a formal radiological tumour assessment was unavailable, thereby constituting a bias, although a sensitivity analysis had been conducted. In the best-case scenario (these 7 patients actually had PD), the PPV was 35/36 (97%). In the worst-case scenario (none actually had tumour progression), the PPV was 77%. The second limitation of this study is the time biais. The median time to progression appeared longer in cases of progression diagnosed by imaging compared to progression diagnosed by clinical judgment. Nevertheless, in case of clinically suspected PD the physicians anticipated the imaging assessment. This shortened the time to progression.

## Conclusions

Definition of PD is a key-element of the design of current phase II trials. Moreover, time to progression or progression-free survival often replace overall survival in contemporary phase III trials. Time to progression and progression-free survival are partly subjective or subject to bias (measurement error in imaging analysis, non radiographic worsening of the clinical state, impact of other causes of treatment discontinuation…) [6]–[7]. Regarding these problems, we attempt to estimate the risk of overestimate the occurrence of tumour progression by the clinical judgment.

This retrospective study demonstrates that the clinical judgment of PD is highly predictive of real PD according to RECIST (positive predictive value of about 96%). Physicians thus do not overestimate PD.

The diagnosis of PD is of major importance in the design of phase II trials, the step of screening studies that is a critical step for the go/no go decision in the drug development process. Nevertheless, the definition of PD integrates both radiological and clinical assessments. This dual and complex definition that includes a part of clinical subjectivity might interfere with the trial results as well in the context of phase II trials as in the context of phase III trials based on time to progression or progression-free survival. This bias might be likely maximal in case of single-arm open phase II and be likely attenuated in case of double-blind randomized trials

This retrospective study underlines the limits of the current definitions of PD (including RECIST). This needs to be more precisely explored in further studies. Moreover, the current basis of definitions have been set up at the time of classical cytotoxic agents development. We are not sure that these definitions are perfectly suitable for the development of new targeted agent, such as tyrosine kinase inhibitors. At the current time of development of myriads of new agents, new definitions of PD are urgently needed [2].

By default, according to this study, clinical judgment of PD, not confirmed by subsequent imaging, appears to be an acceptable criterion for defining PD in clinical trials.
